# Descriptive Analysis of First-Line Non-Small Cell Lung Cancer Treatment with Pembrolizumab in Tumors Expressing PD-L1 ≥ 50% in Patients Treated in Quebec’s University Teaching Hospitals (DALP-First Study)

**DOI:** 10.3390/curroncol30030247

**Published:** 2023-03-11

**Authors:** Ghislain Bérard, Chantal Guévremont, Nathalie Marcotte, Coleen Schroeder, Nicole Bouchard, Raghu Rajan

**Affiliations:** 1 Centre Intégré Universitaire de Santé et de Services Sociaux de l’Estrie—Centre Hospitalier Universitaire de Sherbrooke (CIUSSS de l’Estrie—CHUS), Sherbrooke, QC J1G 1B1, Canada; nicole.bouchard@usherbrooke.ca; 2 McGill University Health Center (MUHC), Montréal, QC H4A 3J1, Canada; chantal.guevremont@muhc.mcgill.ca (C.G.); coleen.schroeder@muhc.mcgill.ca (C.S.); raghu.rajan.med@ssss.gouv.qc.ca (R.R.); 3 CHU de Québec—Université Laval, Québec, QC G1R 2J6, Canada; nathalie.marcotte@chudequebec.ca

**Keywords:** pembrolizumab, immunotherapy, anti-PD-1, non-small cell lung cancer, real-world data

## Abstract

Since July 2017, pembrolizumab has been approved as a first-line treatment of metastatic non-small cell lung cancer (NSCLC) in patients with a PD-L1 score ≥ 50% in Quebec. Study objectives were to describe and assess the real-world use of pembrolizumab; report progression-free survival (PFS), overall survival (OS), and immune-related adverse events (IRAEs); and compare outcomes between a fixed dose (FD) and a weight-based capped dose (WCD). Medical records of patients treated in one of Quebec’s four adult university teaching hospitals who received pembrolizumab between 1 November 2017 and 31 October 2019 were reviewed and followed until 29 February 2020. Two hundred and seventy-nine patients were included. The median real-world PFS and OS were 9.4 (95% CI, 6.6 to 11.2) and 17.3 months (95% CI, 12.9 to not reached), respectively. IRAEs causing delays or treatment interruptions were seen in 34.4% of patients. Initiating treatment with a FD (49 patients) or using a WCD (230 patients) does not appear to affect PFS, OS, or the occurrence of IRAEs. The use of a WCD strategy allowed approximately CAD 5.8 million in savings during the course of our study. These findings support the effectiveness and safety of pembrolizumab in a real-world setting. The use of a WCD does not appear to have a negative impact on patient outcomes.

## 1. Introduction

Lung cancer is one of the most prevalent cancers worldwide as well as the deadliest. In 2020, according to the World Health Organization (WHO), lung cancer ranked first in terms of mortality, with 1.796 million estimated deaths per year (male = 1.188 million; female = 0.6 million), and was ranked second in incidence, with 2.2 million estimated new cases per year (male = 1.435 million; female = 0.7 million), accounting for 11.4% of all new cancer cases worldwide [1].

In Quebec, the number of new patients with lung cancer is estimated at 9000 each year. Non-small cell lung cancer (NSCLC) is the most common type of thoracic cancers [2]. Nearly half of these patients are diagnosed when the disease is already at an advanced stage, most with metastatic disease. A significant number of patients diagnosed at an earlier stage will also eventually progress to metastatic disease. Of those patients who progress, approximately 25% will have tumor PD-L1 levels of 50% or greater [3,4].

In the early 2000s, patients with advanced NSCLC (unresectable or metastatic disease) had a median survival of 8 to 12 months [5,6]. The 5-year survival of patients for all lung cancers combined was approximately 15%, while it was a maximum of 10% for stage IVA cancers and close to zero for stage IVB cancers [7]. Since that time, we have seen an increase in the number of new and effective therapeutic options which have considerably improved clinical results, giving hope for a reduction in mortality in this population [8,9,10].

Since the publication in November 2016 of the KEYNOTE-024 study [11], pembrolizumab has been accepted as a first-line treatment for metastatic NSCLC in patients whose tumor expresses a PD-L1 level of 50% or more. The efficacy and safety data currently available for pembrolizumab in this indication come from two phase III studies, KEYNOTE-024 and KEYNOTE-042 [12], and from a few observational studies. Since the inclusion and exclusion criteria for these studies are quite restrictives, patients found in these pivotal trials may differ from those treated in a real-world setting. In our institutions, an initial collection of data was carried out during the summer of 2018 for all patients who received nivolumab and pembrolizumab between January 2011 and October 2017 [13]. Only 25 patients included in this previous descriptive analysis had received pembrolizumab as a first-line treatment in NSCLC, since reimbursement for this indication in Quebec was only authorized in July 2017. This descriptive analysis will provide a portrait of the use of pembrolizumab as a first-line treatment in metastatic NSCLC in four Quebec adult university teaching hospitals in a real-world context.

It is important to consider the budgetary implications of this treatment for hospital pharmacy departments in Quebec as pembrolizumab is associated with a significant increase in cost relative to current chemotherapy regimens. Since the publication of the KEYNOTE-024 trial, the product monograph for pembrolizumab recommends the use of a fixed dose of 200 mg for all patients. This corresponds to the dose for a patient who weighs 100 kg (2 mg/kg) (the 2 mg/kg was the originally approved dose in melanoma and second-line treatment for lung cancer), which would cost CAD 8800 per dose every 3 weeks. Goldstein et al. published an article in March 2017 that estimated that the use of a personalized weight-based dose in first-line NSCLC would reduce the expenditure of pembrolizumab by approximately 24% in the United States compared to the recommended fixed dose from the FDA-approved monograph [14]. In the fall of 2018, Canada’s Drug and Health Technology Agency (CADTH) and the Programme de Gestion Thérapeutique des Médicaments (PGTM) both recommended the use of 2 mg/kg up to a maximum dose of 200 mg every 3 weeks (henceforth referred to as weight-based capped dose) for pembrolizumab in all indications, including first-line treatment of NSCLC [15,16]. This measure was gradually implemented in Quebec’s oncology treatment centers between summer 2017 and 2019.

The purpose of this study was to investigate the real-world utilization of pembrolizumab and assess real-world progression-free survival (PFS) and overall survival (OS) in patients with advanced NSCLC in our centers. We also wished to confirm that the recommendations made by the PGTM concerning pembrolizumab weight-based capped dosing did not have a negative impact on our patient population.

## 2. Materials and Methods

### 2.1. Study Design and Patients

The descriptive analysis of first-line non-small cell lung cancer treatment with pembrolizumab in tumors expressing PD-L1 ≥ 50% in patients treated in Quebec’s university teaching hospitals (DALP-first study) is a noninterventional, retrospective study that was conducted in four university teaching hospitals in Quebec. All patients 18 years or older that initiated treatment and received at least one dose of pembrolizumab monotherapy as a first-line treatment of a histologically proven, advanced (stage III) or metastatic (stage IV) NSCLC between 1 November 2017 and 31 October 2019 were reviewed. Patients were excluded if they had previously received treatment with an EGFR or ALK inhibitor, if they received pembrolizumab as part of an immuno-chemotherapy combination, or if pembrolizumab was given as part of a clinical trial. Information found in patients’ files of all eligible patients was entered on a standardized data collection sheet. Follow-up was initially planned until 30 June 2020 but was cut to 29 February 2020 because of the global SARS-COVID pandemic in March 2020.

### 2.2. Data Collection

Patient data were obtained retrospectively from medical files and included demographics, characteristics of NSCLC, number and localization of metastatic sites, tumor response to pembrolizumab and toxicities. Histology, PD-L1 score, and genetic abnormalities were evaluated locally in each center.

### 2.3. Ethical Considerations

The study was approved by the Comité d’éthique à la recherche du CIUSSS de l’Estrie—CHUS.

### 2.4. Study Measures

Real-world progression-free survival (PFS) was the primary endpoint, defined as the time from initiation of pembrolizumab to the date of radiologic disease progression or death. As this was a real-world analysis, imaging was carried out at the physician’s discretion. Secondary endpoints included overall survival (OS), defined as the time from treatment initiation to death from any cause, and safety. Adverse events (AE) were graded by each participating center according to the National Cancer Institute Common Terminology Criteria for Adverse Events (CTCAE), version 4.0.

### 2.5. Statistical Analysis

This is a descriptive study for which no theoretical calculation of the number of patients to be included was made. Categorical variables were described as frequencies and percentages. Continuous variables were presented as means (standard deviations) if normally distributed or as median (interquartile range) otherwise. Missing data were only reported and multiple imputation was not performed.

As the main endpoints, both real-world PFS and OS were first estimated using Kaplan–Meier curves. When possible, the median survival time was presented. The log-rank test was used to compare the survival time of subgroups. Age, sex, brain metastases at baseline, Eastern Cooperative Oncology Group (ECOG) performance status, and dose received (fixed vs weight-based capped) were also included as covariates in a Cox model. Results are presented as non-adjusted hazard ratios (HR) and adjusted hazard ratios (aHR) with their 95% confidence interval.

Planned subgroup analyses included a comparison of patients who received a fixed dose to those who received a weight-based capped dose and of patients with ECOG 0–1 vs. ECOG > 1 to ≤2 vs. ECOG > 2. 

Characteristics of patients with early discontinuation was an exploratory endpoint. 

Results were obtained with R software (v4.3.3) (2021), using survival and survminer packages. We considered a level of 5% as significant.

## 3. Results

### 3.1. Characteristics of Patients at Diagnosis of NSCLC

We identified 279 patients with advanced NSCLC who received their first pembrolizumab dose between 1 November 2017 and 31 October 2019. All 279 were included in the analysis.

Patients’ main characteristics are described in Table 1. Overall, 98.9% had a PD-L1 ≥ 50%, 87.5% were metastatic, and 12.5% had a locally unresectable cancer. At the time of treatment initiation, while most patients had an appropriate ECOG score, it was greater than 1 in 18.4% of patients and missing in 6.1% of patients (approved only for patients with an ECOG PS 0–1 in Quebec). In addition, 22.6% had brain metastases; 52 of these 63 patients had metastases that had been treated and were considered stable.

### 3.2. Efficacy

#### 3.2.1. Treatment

At the time of data cut-off on 29 February 2020, the median follow-up was 7.53 months (range, 0.03–26.84). During the study period, the median number of cycles received was 6 (interquartile range 2 to 13). Patients received a minimum of 1 cycle and a maximum of 36 cycles. It should be noted that 4 patients received pembrolizumab for more than 24 months; only one received 36 cycles, exceeding the maximum number of 35 3-week cycles allowed in the original study and which has been approved for reimbursement in Quebec.

Following the CADTH and PGTM recommendations in 2018 [15,16] (and the subsequent INESSS [Quebec’s provincial drug evaluation and health-technology assessments agency] guidance) [17], 230 patients (82.4% of the population) started their treatment using the pembrolizumab weight-based capped dose (2 mg/kg up to a maximum dose of 200 mg) and 49 patients (17.6%) received the 200 mg dose (fixed dose) as recommended by the product monograph. Of the 49 patients who started on the fixed dose, 13 patients were changed to the weight-based capped dose at a later point during their treatment, while 34 patients remained on the fixed dose throughout their treatment. Two of the patients who started on the fixed dose received only one cycle. The number of cycles received before switching to the weight-based capped dose ranged from 1 to 30. No patient who initiated treatment with a weight-based capped dose switched to the fixed dose and thirty of these patients received only one cycle.

#### 3.2.2. Progression-Free Survival and Overall Survival

**Figure 1:**
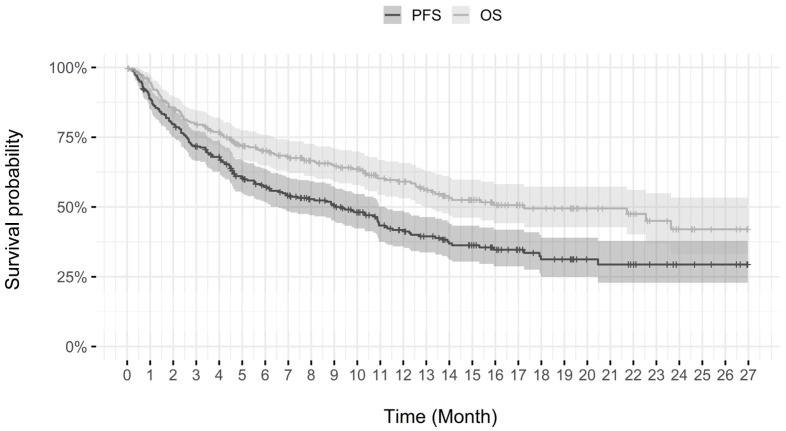
Progression-free survival and overall survival of the study population (*n* = 279).

At the end of the follow-up period on 29 February 2020, 76 patients (27.2%) were still receiving ongoing treatment with pembrolizumab. The median PFS and OS were 9.4 months (95% CI, 6.6 to 11.2) and 17.3 months (95% CI, 12.9 to not reached), respectively (Figure 1).

At 6, 12, and 24 months, the estimated percentages of patients who had no disease progression and were still alive were 57.4% and 70.1% at 6 months, 41.7% and 59.1% at 12 months, and 29.4% and 42.0% at 24 months, respectively.

A Cox regression was performed to determine if any of the variables presented by the patients could have an impact on the response to treatment or the patient’s survival, namely: gender, age, weight, ECOG PS score at treatment initiation (less than or equal to one vs. greater than one and less than or equal to two vs. greater than two), presence of untreated or unstable brain metastases, and presence or absence of an autoimmune disease. It was also possible to look at whether receiving the fixed dose or the weight-based capped dose had an impact on OS.

Utilizing the Cox regression model, patients taking the weight-based capped dose had a HR for death of 0.97 compared to the fixed dose (*p* = 0.88), not considered statistically significant. With adjustment for various confounding factors (gender, ECOG PS, and autoimmune disease), this effect was still found to be non-significant.

Furthermore, it was observed that an ECOG PS greater than two, the presence of untreated or unstable brain metastases at the time of initiating treatment, as well as a weight of less than 50 kg, were predictive of an unfavorable outcome. These patients were treated for a statistically significantly shorter time than the other patients in the study population and also died earlier (Table 2). (Kaplan-Meier curves of survival probability according to ECOG PS score at treatment initiation can be seen in the Supplementary Figure S1.)

### 3.3. Adverse Events

During treatment, immune-related adverse events (IRAEs) causing delays or interruptions in treatment occurred in 27.6% of the patients (77 patients experienced 96 IRAEs). Grade 3–4 IRAEs occurred in 25 patients (9%) (half of which were colitis or other gastro-intestinal adverse events). IRAEs and other side effects of treatment were the main reasons for treatment discontinuation in 44 patients (15.8%). The median delay between the first administration of pembrolizumab and occurrence of a first IRAEs causing a treatment delay or an interruption in treatment was 15.4 weeks.

Patients with a history of autoimmune disease were more likely to experience an IRAE causing a treatment delay or an interruption (44% [11 of 25 patients with autoimmune disease] vs. 26% [66 of 254 patients with no history of autoimmune disease]).

### 3.4. Subgroup Analysis

#### 3.4.1. Dosing Modality

**Figure 2:**
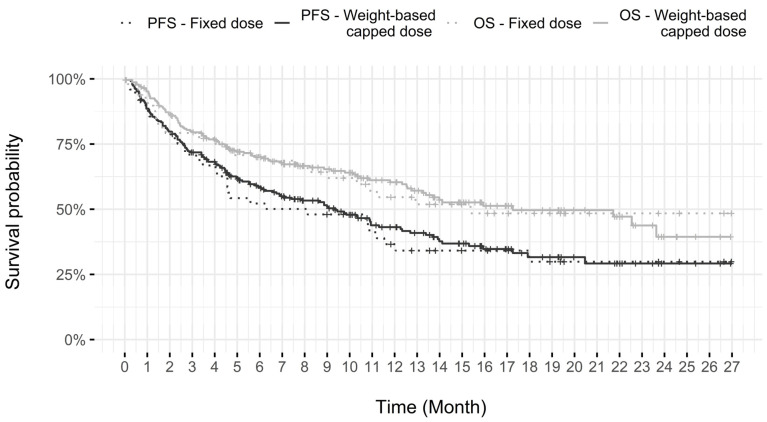
Progression-free survival and overall survival of the study population with regards to pembrolizumab dose used (*n* = 279).

As presented in the Cox regression model and as seen in Table 3 and Figure 2, initiating treatment at a fixed dose of 200 mg or using the weight-based capped dose does not appear to have had an impact on patients’ PFS or OS; the Kaplan–Meier survival curves were more or less superimposable. The dosing strategy used also did not have an impact on the presence of IRAEs, where 28.6% of patients (14 of 49 patients) that started with a fixed dose and 27.4% (63 of 230 patients) on a weight-based capped dose experienced an IREA that caused a delay or interruption in their treatment. Finally, the number of cycles received during their treatment was also similar regardless of the dose modality used at the start of the treatments (Table 3).

#### 3.4.2. Early Treatment Discontinuation (ETD)

A total of 113 patients (40.5% of the population) received 4 or fewer cycles of pembrolizumab (administered every three weeks), equivalent to less than 12 weeks of treatment. They were considered to be in the group of early treatment discontinuation (ETD). Given the difference between the end of the recruitment period (31 December 2019) and the end of the observation period (29 February 2020), most patients who received four cycles or less of pembrolizumab had indeed stopped their treatment with pembrolizumab. Only one of these patients was still on treatment at the end of the observation period, as he had had a delay of 2.3 months in the administration of his cycles due to an IRAE that occurred early in treatment (three weeks after treatment initiation).

At the time of data cut-off, 83 patients (73.5%) in this group had died and 24 (21.2%) were still alive, with 1 patient still on treatment, and 6 were lost to follow-up. Comparatively, in the non-ETD group, there were 35 deaths (21.1%), 128 patients (77.1%) were still alive (75 [45.2%] were still on treatment), and 3 were lost to follow-up.

By comparing the characteristics of ETD patients, as demonstrated by the Cox regression (Table 2), we observed that the two biggest predictors of ETD were an ECOG PS higher than two as well as unstable or untreated brain metastases. In fact, 11 of 14 patients who had an ECOG PS greater than 2 and 7 of 10 that had unstable or untreated brain metastases were in the ETD group. To these characteristics, we can also add the presence of an autoimmune disease (15 of the 25 patients who had an autoimmune disease), as well as weighing less than 50 kg (21 of 33 patients weighing less than 50 kg).

**Figure 3:**
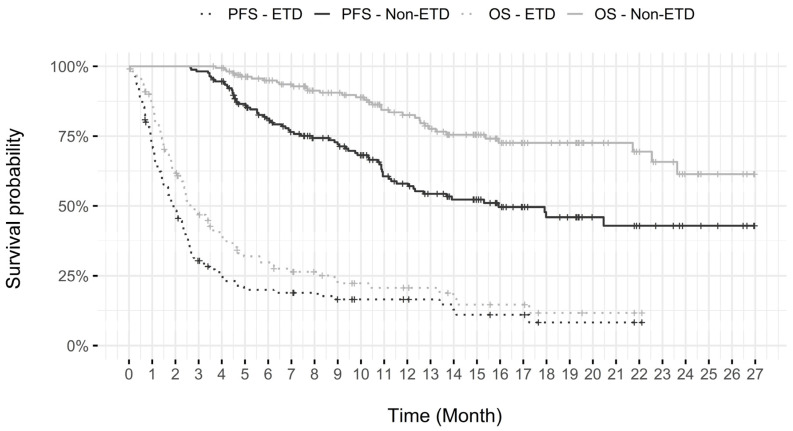
Survival probability of ETD vs. non-ETD.

As seen in Table 4, the rate of patients that discontinued treatment for progression was similar between the non-ETD and the ETD group, although PFS and OS were shorter in the ETD group, as seen in Figure 3. However, 45 of 52 patients (86.5%) that either died within 3 weeks of the last pembrolizumab dose, voluntarily stopped their treatment, or discontinued treatment for another reason (including transfer to palliative care without clear progression) were in the ETD group. The percentage of patients that discontinued treatment for IRAEs or other side effects was also higher in the ETD group (18.6% vs. 13.9%).

## 4. Discussion

This real-world, retrospective, observational study evaluating pembrolizumab as a first-line treatment for advanced NSCLC patients with a PD-L1 score ≥ 50% and without sensitizing EGFR mutations or ALK translocations, showed a PFS of 9.4 months (95% CI, 6.6 to 11.2). At the time of data cut-off, 42.3% of the enrolled patients (118 of 279) had died with a median OS of 17.3 months (95% CI, 12.9 to not reached) and a relatively low frequency of treatment-related IRAEs was observed. The real-world PFS finding of 9.4 months is consistent with those of pembrolizumab in the pivotal phase 3 KEYNOTE-024 trial (10.3 months) and in the subgroup of previously untreated NSCLC with PD-L1 score ≥ 1% in KEYNOTE-042 trial (7.1 months) [11,12], noting the shorter follow-up in this descriptive analysis. However, the OS of 17.3 months was shorter than seen in KEYNOTE-024 (30 months) but closer to that of the KEYNOTE-042 subgroup (20.0 months). It is important to note that this descriptive analysis was a retrospective analysis based on an unselected, real-world patient population including 18.8% of patients with an ECOG PS of 2 or higher (ECOG PS = 2 in 24 patients and >2 in 14 patients) and 6.1% (17 patients) where ECOG PS was not documented in the medical file at the time of treatment initiation. In comparison, the patients enrolled in the KEYNOTE-024 trial were highly selected: out of a total of 1653 patients whose tumors could be evaluated for PD-L1 score, 500 patients were ≥50% but only 300 of these were included in the study. Further analysis of our data suggests that if only the patients responding to the KEYNOTE-024 inclusion criteria would have received pembrolizumab, the PFS would have been 10.5 months and OS 21.7 months, which would have been more in line with the results of the pivotal study (see Supplementary Figure S2). In addition, the OS rate in our study differed from that of KEYNOTE-024 after a follow-up of 6 months (86.2% vs. 70.1%), potentially due to the fact that nearly 40% of our population received four cycles (approx. 12 weeks) or less of pembrolizumab.

Our results are quite similar to those of a real-world cohort study in a French population (PEMBREIZH) [18] (Table 5).

As seen in the Cox regression (Table 2), an ECOG PS greater than two and the presence of untreated or unstable brain metastases at the time of initiating treatment were predictive of an unfavorable outcome. Both of these variables were more prevalent in our review than in the two pivotal trials. We also observed in the Cox regression that a weight of less than 50 kg could have a negative impact on survival outcome. This analysis was added after the publication of Pasello et al. who found that a body mass index (BMI) of less than 25 is a predictor of early treatment discontinuation. [19] As the height was not collected in our study, a weight of less than 50 kg was chosen as a surrogate for a low BMI which may characterize a more fragile or frail patient population (in our population, 33 of 279 patients (11.8%) weighed less than 50 kg and 21 of them received 4 cycles of pembrolizumab or less). Clearly, not all patients weighing less than 50 kg can be considered frail; however, this is another factor to consider when determining whether to use pembrolizumab.

In this analysis, the rate of IRAEs of any grade was low (34.4%) compared to the rates reported on KEYNOTE-024 and KEYNOTE-042 (73.4% vs. 63%, respectively) but we only accounted for IRAEs that caused delays or treatment interruptions. IRAEs were actually responsible for 15.8% of definitive drug discontinuation (44 of 279 patients) in this analysis compared to 7.1% and 9% in the pivotal studies. This may be explained by patient selection in the pivotal trials which is generally more strict than in a real-life setting; our population was older, had a higher ECOG PS score and more patients had a history of autoimmune disease at treatment initiation.

When looking at patients that discontinued pembrolizumab early (the ETD group in Table 4), some of what was observed might be explained by patient selection. Further studies might be needed to help identify whether early discontinuation is associated with certain subsets of these patients, for example, patients with rapidly evolving cancers. In these patients, the effect of immunotherapy on the immune system and on the cancer might be delayed and cannot overcome the rapid progression of the tumor.

Concerning the cost of treatment, two key points were considered. First of all, as concluded by Freshwater et al., “doses of 200 mg and 2 mg/kg provide similar exposure distributions with no advantage to either dosing approach (…). These findings suggest that weight-based and fixed-dose regimens are appropriate for pembrolizumab” [20]. Secondly, Goldstein et al. were the first to claim that: “Personalized dosing of pembrolizumab may have the potential to save approximately $0.825 billion annually in the United States (24% of pembrolizumab cost), likely without impacting outcomes. This option should be considered for the first-line management of PD-L1-positive advanced lung cancer” [14]. With this in mind, this descriptive analysis did not find any major differences in treatment outcomes (PFS, OS, IRAE, median number of cycles) between patients treated with a weight-based capped dose and those that received a fixed pembrolizumab dose. The results were in line with other pembrolizumab trials in this setting and the use of a weight-based capped strategy has allowed our four university teaching hospitals to save a total of approximately CAD 5.8 million during the course of our study. This amount accounts for 26% of what would have been spent if a fixed dose of pembrolizumab had been given to every patient (CAD 16,462,800 instead of CAD 22,255,200 if every patient had received the fixed dose). If all of the 279 patients had received the weight-based capped dose, the savings would have been nearly CAD 1 million more, representing 30% of the total cost, with efficacy results similar to those seen in the pivotal studies.

## 5. Conclusions

The results of KEYNOTE-024 have changed the landscape for advanced NSCLC patients with a PD-L1 score ≥ 50%. Monotherapy with pembrolizumab, and more recently with cemiplimab, is now the standard of care for first-line treatment of this population. The findings of this study support the effectiveness and safety of pembrolizumab in a real-world cohort of unselected advanced NSCLC patients with a PD-L1 score ≥ 50%. The decision to start pembrolizumab in patients with an ECOG PS > 2 or with untreated brain metastasis should be weighed against the potential risks (including financial risk) of the treatment. Our analysis also shows that the use of a weight-based capped dose with pembrolizumab does not have a negative impact on patient outcomes and can optimize the use of precious financial resources for healthcare systems in a time of escalating oncology drug costs. Since the data collection for this descriptive analysis, a six-weekly dose of pembrolizumab has been incorporated into NSCLC therapy, and other checkpoint inhibitors (including but not limited to cemiplimab, nivolumab, avelumab, dostarlimab, and PD1-Vaxx) are being tested. The results of this study will need to be evaluated with regard to these new therapies. As time passes, more and more patients will stop pembrolizumab after the first two years of treatment and will possibly need to be re-exposed to pembrolizumab after a certain period of time. Real-world data will be of utmost importance to understand how these patients respond to a second course of immunotherapy.

## Figures and Tables

**Table 1:** Patient characteristics (*n* = 279) at treatment initiation with pembrolizumab.

Gender		Number of Patients	%
Men		129	46.2
Women		150	53.8
**Age**			
Mean ± standard deviation		68.1 ± 8.5	
Median		68	
Range (min–max)		34–94	
Interquartile range		62–74	
Patients ≥ 70 years		123	44.1%
**Weight (kg)**			
Mean ± standard deviation		68.3 ± 16.6	
Median		67	
Range (min–max)		32–127	
Interquartile range		56–80	
**ECOG PS score**		**Number of patients**	**%**
0–1		211	75.7
>1–≤2		37	13.3
>2		14	5.0
Unknown/NFF		17	6.1
**Autoimmune disease**			
Yes		25	9.0
No		254	91.0
**Lung cancer staging**			
Metastatic		244	87.5
Advanced (stage III)		35	12.5
**PD-L1 Score**			
Positive ≥ 50%		276	98.9
Positive, but < 50%		2	0.7
Negative		0	-
Unknown/NFF		1	0.4
**Brain metastases**			
Absence		197	70.6
Presence		63	22.6
	Treated and stable	52	18.6
	Untreated and/or unstable	10	3.6
	Unknown if treated or not	1	0.4
Unknown/NFF		19	6.8

Abbreviation: NFF—not found in file.

**Table 2:** Univariate Cox regression and impact on overall survival.

Variables *		HR	95% Confidence Interval		*p*-Value
			Lower Limit	Upper Limit	
Dose	Fixed vs. weight-based capped dose (*n* = 49 vs. *n* = 230)	0.97	0.61	1.53	0.88
Age	Less than 70 vs. 70 or more (*n* = 156 vs. *n* = 123)	1.02	0.71	1.47	0.91
Sex	Men vs. women (*n* = 129 vs. *n* = 150)	1.02	0.71	1.47	0.92
Weight	50 kg or more vs. less than 50 kg (*n* = 246 vs. *n* = 33)	2.61	1.65	4.13	<0.0001
ECOG PS > 2 (*n* = 262)	2 or less vs. more than 2 (*n* = 248 vs. *n* = 14)	4.11	2.13	7.93	<0.0001
ECOG PS > 1 (*n* =262)	1 or less vs. more than 1 (*n* = 211 vs. *n* = 51)	1.34	0.77	2.34	0.07
Brain metastases (*n* = 259)	None or treated vs. untreated/unstable (*n* = 249 vs. *n* = 10)	2.18	1.01	4.7	0.047
Autoimmune disease	No vs. yes (*n* = 254 vs. *n* = 25)	1.34	0.77	2.34	0.31

* if not specified (*n* = 279).

**Table 3:** Number of pembrolizumab cycles received as of 29 February 2020 as per dosing strategy.

Number of Cycles Received	Fixed Dose (*n* = 49)	Weight-Based Capped Dose (*n* = 230)
Mean ± standard deviation	9.3 ± 9.7	9.0 ± 8.2
Median	5	6
Range (min–max)	1–36	1–34
Interquartile range	2–14	2.25–13

**Table 4:** Reasons for discontinuing pembrolizumab treatment.

		Non-ETD Population (More than 4 Cycles of Pembrolizumab) (*n* = 166)		ETD Population (4 Cycles of Pembrolizumab or Less) (*n* = 113)	
		**Number of patients**	**%**	**Number of patients**	**%**
Still receiving initial treatment at data cut-off		75	45.2	1	0.9
Discontinued Treatment		91	54.8	112	99.1
Reason of discontinuation					
	Progressive disease	58	34.9	42	37.2
	IRAE or other side effect	23	13.9	21	18.6
	Death less than 3 weeks after last dose	1	0.6	24	21.2
	Patient decision	1	0.6	12	10.6
	Complete response to treatment	1	0.6	0	-
	Other	5	3.0	9	8.0
	Loss to follow-up	2	1.2	4	3.5

Abreviations: ETD—Early treatment discontinuation; IRAE—Immune related adverse event.

**Table 5:** Comparison of PFS, OS, and IRAE of PGTM population with those of other studies on pembrolizumab as a first-line treatment of NSCLC in patients with a PD-L1 ≥ 50%.

		PGTM	Pembreizh	KEYNOTE—024	KEYNOTE—042 (Subgroup of Patients with PD-L1 ≥ 50%)
Number of patients		279	108	154	299
Median age (years)		68	67	64.5	63
ECOG PS 0–1		75.7%	76.9%	99.4%	100%
Brain metastases		22.6%	17.6%	11.7%	6%
	Unstable or untreated	3.6%	NA	0%	0%
Median follow-up (months) (range)		7.5 (0.03 *–26.8)	8.2 (0.9–20.9)	11.2	12.8
PFS (months) (95% CI)		9.4 (6.6–11.2)	10.1 (8.8–NR)	10.3 (6.7–NR)	7.1 (5.9–9.0)
PFS at 6 months		57.4%	62.7%	62.1%	≈53%
OS (months) (95% CI)		17.3 (12.9–NR)	15.2 (13.9–NR)	30.0 (18.3–NR)	20.0 (15.4–24.9)
OS at 6 months		70.1%	86.2%	80.2%	≈74%
IRAE (All grade)		34.4% **	46.3%	73.4%	63%
IRAE (Grade 3–4)		8.6%	8%	9.7%	8%

Abbreviations: ECOG PS—Eastern Cooperative Oncology Group performance status, CI—confidence interval, PGTM—programme de gestion thérapeutique des médicaments, NR—not reached, NA—not available, IRAE—immune related adverse event, OS—overall survival, PFS—progression-free survival. * One patient died the day after receiving his first dose of pembrolizumab; ** only IRAE causing delays or treatment interruptions were considered.

## Data Availability

The data presented in this study are available upon request from the corresponding author. The data are not publicly available due to personal data protection and privacy of health information.

## Supplementary Materials

The following supporting information can be downloaded at: https://www.mdpi.com/article/10.3390/curroncol30030247/s1, Figure S1: survival probability according to ECOG PS score at treatment initiation. Figure S2: survival probability in patients corresponding to all of Quebec’s reimbursement criteria for pembrolizumab in first-line NCSLC.
