# Supplementary material for: Descriptive Analysis of First-Line Non-Small Cell Lung Cancer Treatment with Pembrolizumab in Tumors Expressing PD-L1 ≥ 50% in Patients Treated in Quebec’s University Teaching Hospitals (DALP-First Study)
Source: Curr Oncol. 2023 Mar 11;30(3):3251–62. doi: 10.3390/curroncol30030247 (PMC10047395; doi:10.3390/curroncol30030247)
Supplement: Supplementary file 1 [file curroncol-30-00247-s001.zip › curroncol-2225759-supplementary.pdf]

Current Oncology

Supplementary Materials:

Figure S1: Survival probability according to ECOG PS score at treatment initiation.

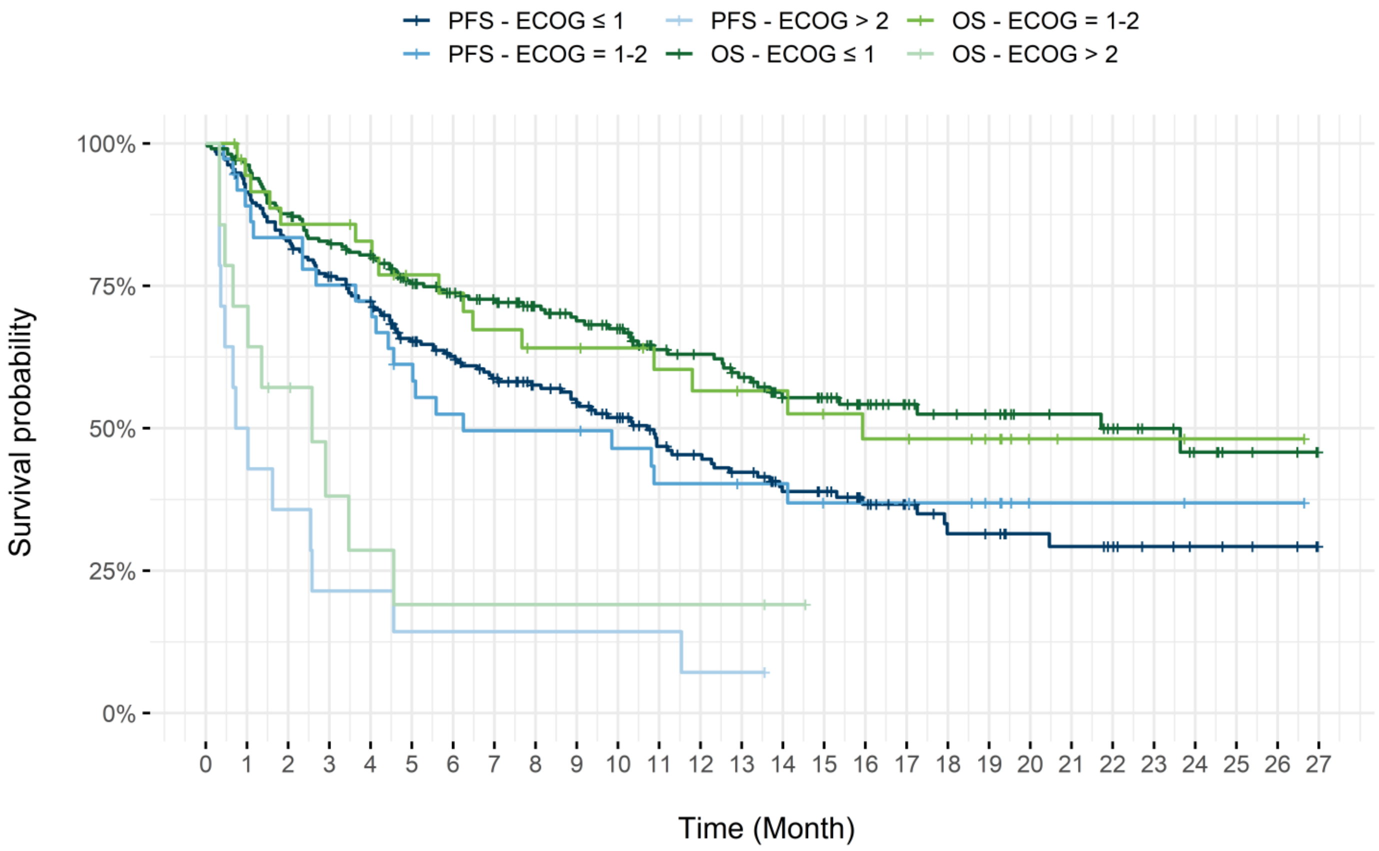

Figure S2: Survival probability in patients corresponding to all of Québec's reimbursement criteria for pembrolizumab in first-line NCSLC

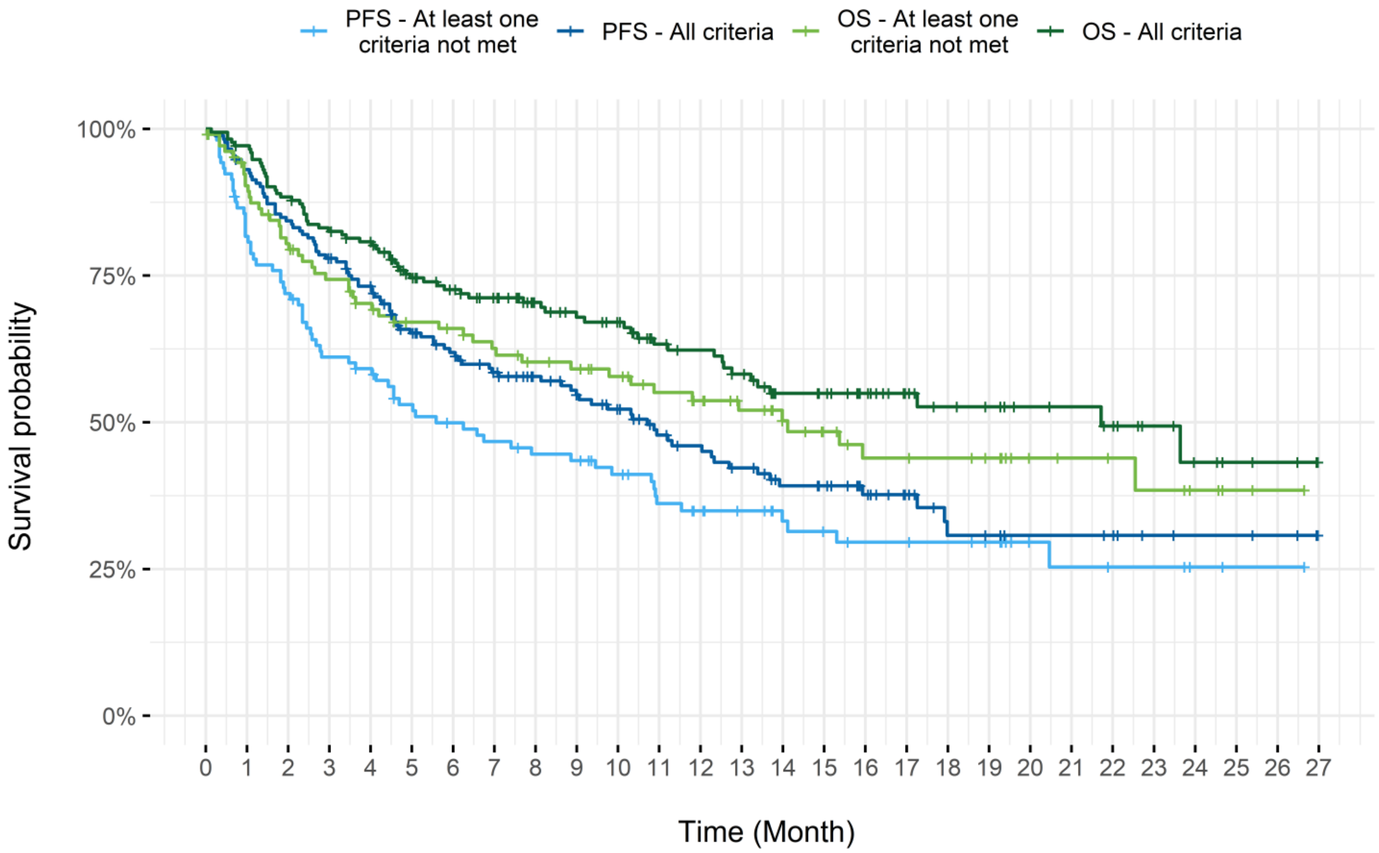

In Québec, for pembrolizumab to be reimbursed for NSCLC, it must be used as monotherapy, for first-line treatment of non-small cell lung cancer at the metastatic stage, in persons:

• whose tumor does not show an activating mutation of the EGFR tyrosine kinase or a rearrangement of the ALK gene;

and

• whose percentage of tumor cells expressing PD-L1 is at least 50%;

and

• whose ECOG performance status is 0 or 1;

and

• whose central nervous system metastases, if present, are treated and stable.

Patients corresponding to all these criteria were included in the "all criteria" group. If one or more of these were not met, patients were placed in "At least one criteria not met".
